# Supplementary material for: Deep Learning Assisted Diagnosis of Onychomycosis on Whole-Slide Images
Source: J Fungi (Basel). 2022 Aug 28;8(9):912. doi: 10.3390/jof8090912 (PMC9504700; doi:10.3390/jof8090912)
Supplement: Supplementary file 1 [file jof-08-00912-s001.zip › jof-1845376-supplementary.pdf]

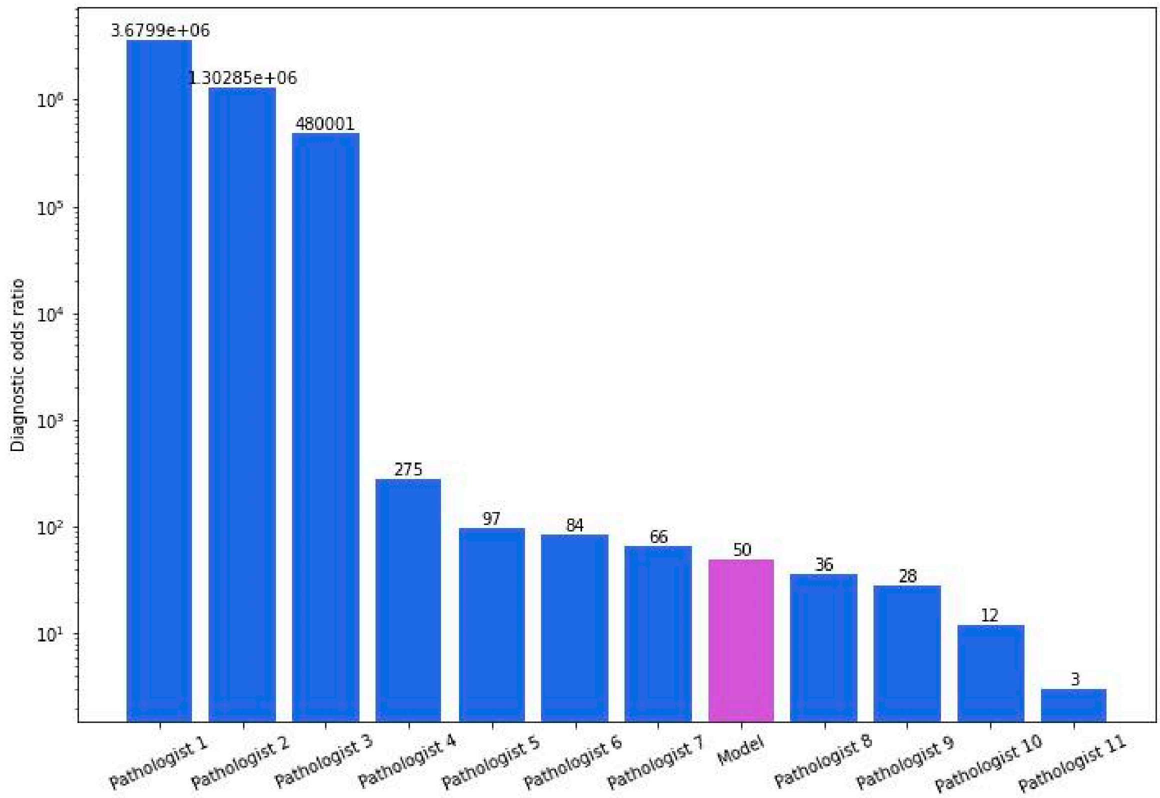

Supplemental Figure S1. Diagnostic odd ratio (DOR) for each pathologist and for the model. The DOR represents the ratio of odds of the diagnosis being positive if the patient has a disease, relative to the odds of the diagnosis being positive if the patient does not have the disease. As two of our pathologists had zero false negatives, we added 0.0001 to every cell of the confusion matrix for every pathologist and the model, to avoid an undefined result. Our model performs in line with the participating pathologists.
